# Radiation therapy for prostate cancer in Syrian refugees: facing the need for change

**DOI:** 10.3389/fpubh.2023.1172864

**Published:** 2023-05-31

**Authors:** Mehmet Fuat Eren, Sarah S. Kilic, Ayfer Ay Eren, Sedenay Oskeroglu Kaplan, Fatma Teke, Tugce Kutuk, Beyhan Ceylaner Bicakci, Lara Hathout, Shalini Moningi, Peter Orio, Banu Atalar, Mutlay Sayan

**Affiliations:** ^1^Marmara University Istanbul Pendik Education and Research Hospital, Istanbul, Türkiye; ^2^Taussig Cancer Institute, Cancer Center, Cleveland Clinic, Cleveland, OH, United States; ^3^Istanbul Kartal Dr.Lutfi Kirdar Education and Research Hospital, Istanbul, Türkiye; ^4^Şanlıurfa Mehmet Akif İnan Eğitim ve Araştırma Hastanesi, Sanliurfa, Türkiye; ^5^Dicle University, Diyarbakır, Türkiye; ^6^Malatya Education and Research Hospital, Malatya, Türkiye; ^7^Rutgers Cancer Institute of New Jersey, The State University of New Jersey, New Brunswick, NJ, United States; ^8^Dana-Farber Cancer Institute and Brigham and Women’s Hospital, Harvard Medical School, Boston, MA, United States; ^9^Acibadem Maslak Hospital, Istanbul, Türkiye

**Keywords:** prostate cancer, global health, refugee, radiation therapy, Syria, Turkey

## Abstract

**Purpose:**

To report the utilization of radiation therapy in Syrian refugee patients with prostate cancer residing in Turkey.

**Methods and materials:**

A multi-institutional retrospective review including 14 cancer centers in Turkey was conducted to include 137 Syrian refugee patients with prostate cancer treated with radiation therapy (RT). Toxicity data was scored using the National Cancer Institute Common Terminology Criteria for Adverse Events version 3.0. Noncompliance was defined as a patient missing two or more scheduled RT appointments.

**Results:**

Advanced disease, defined as stage III or IV, was reported in 64.2% of patients while androgen deprivation therapy (ADT) was only administrated to 20% of patients. Conventionally fractionated RT with a median number of 44 fractions was delivered to all patients with curative intent (*n* = 61) while palliative RT (*n* = 76) was delivered with a median number of 10 fractions. The acute grade 3–4 toxicity rate for the entire cohort was 16%. Noncompliance rate was 42%.

**Conclusion:**

Most Syrian refugee prostate cancer patients presented with advanced disease however ADT was seldom used. Despite the low treatment compliance rate, conventional fractionation was used in all patients. Interventions are critically needed to improve screening and increase the use of standard-of-care treatment paradigms, including hypofractionated RT and ADT.

## Introduction

The Syrian refugee crisis, a result of the still-ongoing 2011 Syrian Civil War, is the largest displacement crisis in the modern world. More than half of the Syrian population is currently displaced, with nearly six million Syrians residing as refugees in host countries. Of these, the majority—nearly four million—reside in Turkey (1). In 2013, Turkey committed to free and equitable access to medical care for all refugees with the Law on Foreigners and International Protection, giving refugees the same rights to healthcare as Turkish citizens (2). However, practical implementation of this legislature faces many obstacles. On a population level, hosting refugees has placed significant strain on Turkey’s economy: in 2020, the United Nations High Commissioner for Refugees (UNHCR) budgeted 365 million US dollars for refugees and asylum-seekers in Turkey, but the available funds were only 131 million US dollars, a deficit of 234 million US dollars (3). On an individual patient level, even when healthcare itself is cost-free, language barriers, lower health literacy, limited access to transportation, housing insecurity, and inability to miss work all detract from refugees’ ability to obtain care (4). Furthermore, refugee health initiatives typically focus on communicable diseases and non-communicable chronic diseases with severe acute consequences if untreated (e.g., insulin for patients with type 1 diabetes), meaning that screening, diagnosis, and treatment for other conditions, including cancer, are even more profoundly impacted (5, 6). Therefore, from both a patient-level and a system-level perspective, the importance of delivering efficient medical care to refugees that is both high-quality and high-value cannot be overstated.

As a result of the aforementioned barriers, refugee patients with cancer suffer from poorer outcomes compared to non-refugee patients. Refugee patients with cancer are less likely to undergo screening, present with more advanced cancer at diagnosis, experience more treatment delays and interruptions, and are more likely to have a poorer prognosis than non-refugee patients (7–9). These disparities are of concern to all oncologists, and particularly radiation oncologists, given the burden placed on patients by daily visits for radiation therapy (RT).

Prostate cancer represents a particularly important target for optimization of diagnosis and treatment in refugee patients for several reasons: it is one of the most common malignancies worldwide, it can be easily screened for, and it has multiple similarly-efficacious treatment options (10). Particular attention should be paid to options for RT in refugee patients with prostate cancer. Radiation treatment modalities are of comparable effectiveness but vary widely with regard to socioeconomic and transportation burden on the patient, with options ranging from low-dose-rate brachytherapy, requiring only a single visit, to conventionally fractionated external beam RT, requiring up to 40 or more visits depending on choice of fractionation. Careful selection of a treatment regimen that offers oncologic effectiveness while minimizing financial and logistical burden on the patient is crucial in this vulnerable population. However, current practice patterns in this population are not well-understood. One previous study of radiation therapy utilization and compliance in a refugee population exists, and identified a high rate of noncompliance in this population, but did not report prostate cancer-specific findings (11). Here we present the first study to our knowledge investigating prostate cancer diagnosis and utilization of RT in refugee patients.

## Methods

We performed a retrospective review of the institutional databases of 14 cancer centers in Turkey to identify Syrian refugee patients aged 18 or older with a prostate cancer diagnosis treated with radiation therapy (RT) between January 2015 and December 2019. Each center included in the study was a member of the Turkish national radiation oncology society, Türk Radyasyon Onkolojisi Derneği. Refugee status was ascertained by the presence of the billing identification code for Syrian refugee status in patients’ electronic medical records. Cancer centers included in the study were selected based on regions known to host the largest populations of Syrian refugees based on data from the United Nations High Commissioner for Refugees (12). Demographic, clinical, treatment, toxicity, compliance, and follow-up data were abstracted directly from the official medical record for each patient. Demographic and clinical data abstracted included age, smoking history, family history of cancer, residence type (refugee camp versus house), proximity of residence to the Turkey-Syria border, and oncologic variables including stage, treatment intent, use of androgen deprivation therapy (ADT), and details of radiation therapy treatment. Toxicity data were assessed by the physician at on-treatment visits and were classified based on the National Cancer Institute Common Terminology Criteria for Adverse Events (CTCAE) version 3.0. Noncompliance was assessed as a binary variable based on the validated definition of a patient missing two or more scheduled RT appointments (13). Treatments that were missed due to medical or facility circumstances (i.e., machine malfunction, physician-recommended treatment break) were not scored as noncompliant. Follow-up time was defined as the time from the completion of RT to the most recent follow-up date. Data analysis was performed using SPSS Statistics version 25 (IBM Corp., Armonk, NY, United States). This study was approved by the Institutional Review Boards of Marmara University Pendik Research and Education Hospital (IRB# 09.2019.615) and each individual participating center.

## Results

A total of 10,537 Syrian refugee patients were diagnosed with cancer during the study timeframe. Of these, 137 (1.3%) were diagnosed with prostate cancer and treated with RT. The median age at the time of treatment initiation was 65 years (range, 54–86 years). Thirty-four percent of patients resided in a refugee camp, and 66% resided in a house. Fifty-eight percent of patients resided near the Turkey-Syria border. Sixty-five percent of patients had a history of smoking. The vast majority of patients (98.5%) had no known family history of cancer. The majority of patients (64.3%) were diagnosed with advanced disease, defined as stage III or IV. Twenty percent of patients received ADT. The median follow-up for the cohort was 13 months (range, 4–36 months). Demographic and clinical characteristics of the cohort are displayed in Table 1.

**Table 1:** Demographic and clinical characteristics of study cohort (*n* = 137).

	Median (range)
Age (years)	65 (54–86)
	*n* (%) (Total *n* = 137)
Smoking history	
Yes	89 (65.0)
No	48 (35.0)
Family history of cancer	
Yes	2 (1.5)
No	135 (98.5)
Residence type	
Refugee camp	47 (34.3)
House	90 (65.7)
Residence near Turkey-Syria border	
Yes	80 (58.4)
No	57 (41.6)
Cancer stage	
I	17 (12.4)
II	32 (23.4)
III	12 (8.8)
IV	76 (55.5)
Androgen deprivation therapy	
Yes	27 (19.7)
No	110 (80.3)

Regarding RT, 45% of patients were treated with definitive intent and 55% with palliative intent. In definitive cases, all 61 patients were treated with conventionally-fractionated regimens. The median number of fractions was 44. In palliative cases, all 76 patients were treated with long-course palliative regimens of 10–15 fractions, and the median number of fractions was 10. The acute grade 3–4 toxicity rate for the entire cohort was 16%. There were no grade 5 toxicities. Forty-two percent of patients were noncompliant with treatment based on the definition of two or more missed fractions. RT treatment data are displayed in Table 2. A comparison of treatment characteristics based on residency type is displayed in Table 3. Rates of treatment noncompliance were significantly higher among patients who lived in a refugee camp compared to those who lived in a house (64% vs. 30%, *p* < 0.001).

**Table 2:** Radiation therapy treatment details (*n* = 137).

	*n* (%)
Treatment intent	
Definitive	61 (44.5)
Palliative	76 (55.5)
Grade 3–4 acute toxicity	
Yes	22 (16.1)
No	115 (83.9)
Compliance with treatment	
Yes	80 (58.4)
No	57 (41.6)
	Median (range)
Number of fractions (definitive cases)	44 (34–45)
Number of fractions (palliative cases)	10 (10–15)
Follow-up time (months)	13 (4–36)

**Table 3:** Comparison of treatment characteristics based on residency type (*n* = 137).

	Residence type		
	House *n* (%)	Refugee camp *n* (%)	*p*-value
Treatment intent, *n* (%)			0.453
Definitive	52 (57.8)	24 (51.0)	
Palliative	38 (42.2)	23 (49.0)	
Androgen deprivation therapy, *n* (%)	16 (59.0)	11 (41.0)	0.432
Noncompliance with treatment, *n* (%)	27 (30.0)	30 (63.8)	<0.001
Number of fractions (definitive cases), mean	43.4	43.8	0.885
Number of fractions (palliative cases), mean	10.7	10.5	0.727

## Discussion

In this study, we present the first-ever investigation of prostate cancer diagnosis and utilization of radiation therapy in refugee patients. We identified several concerning trends in this population. Patients were overwhelmingly diagnosed with more advanced disease at diagnosis, did not receive standard-of-care androgen deprivation therapy (ADT), and received logistically inconvenient conventionally-fractionated RT courses. Each of these findings represents important targets for intervention.

Prostate cancer diagnoses were made at an advanced stage in this population: stage IV was the most common stage at diagnosis (56% of patients), and an additional 9% of patients were diagnosed with stage III disease. In contrast, in a large study of prostate cancer epidemiology in the general Middle Eastern population, only 22.6% of patients presented with metastatic disease (14). These findings likely reflect the significant barriers to medical care faced by refugee patients, which contribute in multiple ways to cancer progression. Firstly, as detailed above, cancer screening is severely underutilized in refugee populations. This is a particularly important contributor to advanced stage at diagnosis in prostate cancer, which is frequently asymptomatic for months or years while disease progresses locally and distantly. Secondly, even if refugee patients have symptoms, the obstacles discussed above such as language barriers, lack of access to transportation, and inability to miss work impair this population’s ability to seek medical care when it is needed. All of these factors contribute to delays in cancer diagnoses in refugees and more advanced stage at diagnosis. These findings emphasize the critical need for diversifying health outreach programs aimed at refugees. As discussed above, health outreach programs aimed at migrants and refugees typically focus on infectious diseases and acutely critical conditions. As a result, screening, diagnosis, and treatment of long-term or chronic conditions, such as prostate cancer, fall by the wayside and result in the entirely preventable progression to advanced and metastatic disease in many patients, as demonstrated in the present study. Guidelines for cost-effective prostate specific antigen (PSA)-based screening programs in low- and middle-income nations exist and can be used as a framework for implementing a screening in refugee populations, with principles including interpreting PSA levels according to age, screening less frequently, and increasing prostate cancer awareness in the target population with accompanying education programs (15).

ADT is a standard of care for locally advanced and metastatic prostate cancer worldwide. However, despite the fact that 65% of refugee patients in the present study presented with locally advanced or metastatic disease, only 20% received ADT. This rate of utilization of a widely-acknowledged standard-of-care treatment is alarmingly low and raises the question of why ADT use was so low in this population. Consensus recommendations specific to the Middle East echo the international prostate cancer community’s stance on the importance of ADT for men with advanced disease, suggesting that lack of physician awareness is not likely to be the explanation for low use in the refugee population (16). Furthermore, a survey of Turkish urologists and oncologists demonstrated an overwhelming preference for the use of ADT rather than surgical castration for men with metastatic prostate cancer, suggesting that use of surgical castration instead of ADT is not likely to be the reason for low rates of ADT use in the present study (17). Possible explanations for the low utilization of ADT in this population may include physician hesitance to prescribe ADT in a population perceived as more vulnerable, fragile, or nonadherent to follow-up, and may also include patient factors such as fear of side effects or lack of understanding of importance of the intervention. Use of ADT in the refugee population represents an important target for intervention for several reasons. Firstly, healthcare equity is a crucial component of any global health initiative, including healthcare for migrants and refugees, as defined by the World Health Organization (18). Secondly, ADT is the most cost-effective systemic therapy for prostate cancer in a low-resource setting (19), and can be administered in depot form allowing administrations to happen as infrequently as once every six months. Affordability and convenience of treatment schedule make ADT an ideal intervention for refugee patients who face the barriers to care described above.

With regard to radiation therapy, most patients (76% or 56%) were treated with palliative intent, concordant with our finding that 56% of patients presented with metastatic disease. In all 76 palliative cases, patients were treated with long courses of a minimum of 10 to a maximum of 15 fractions. Similarly, in patients treated with definitive intent, all patients received long-course RT, with a median number of fractions of 44. The noncompliance rate, defined as missing two or more fractions, was 42%. Our findings demonstrate the alarming trend of using ultra-long-course RT regimens in refugee patients (11). In this population of patients who face serious barriers to care and who are already known to have a high rate of treatment noncompliance, offering protracted courses of therapy when equally-effective shorter courses exist creates serious burden for the patient without any clear oncologic benefit. With regard to palliative RT in underserved populations, for uncomplicated bone metastases, which likely represent the majority of treatment courses in the present study, single-fraction palliative RT is the preferred regimen based on clinical effectiveness and cost-effectiveness (20). Radiation oncologists caring for refugee and migrant patients, or other patients with serious barriers to care, should strongly consider single-fraction or fewer-fraction regimens for the palliation of uncomplicated metastases.

Equally concerning was the present study’s finding that all patients treated with definitive intent received protracted, conventionally-fractionated RT courses. Hypofractionated regimens are similar in both efficacy and toxicity to conventionally-fractionated regimens (21) and are supported by consensus guidelines (22). Furthermore, guidelines for prostate cancer RT in developing countries recommend the use of hypofractionated regimens due to favorable cost-effectiveness and improved access to care (23). Despite these well-established standards, no patients in the present study were treated with hypofractionated courses. Given the significant barriers to care and financial, transportation, and employment burden placed on refugee patients by protracted treatment courses, hypofractionated RT would be of particular practical benefit in this population. Increasing the use of hypofractionated RT courses in refugee populations therefore represents a critical target for intervention. Radiation oncologists who care for refugee patients should be educated regarding the value of offering hypofractionated courses in this population. When available, low-dose-rate prostate brachytherapy should be considered a salient option in this population given that it requires only a single visit to the department and is more cost effective than both high-dose-rate brachytherapy and long-course intensity-modulated EBRT (24).

Noncompliance, defined as two or more missed fractions, was high in the present study: 42% of patients were noncompliant. Given that noncompliance is associated with inferior recurrence-free survival and overall survival in cancer patients treated with RT (13), this finding is alarming, particularly in an already vulnerable population. Interventions to improve access to care, and therefore compliance, in refugee patients have been well-studied and include health awareness campaigns in refugees’ native language, interpretive services at healthcare visits, and transportation services (25). Provisioning of funds from host countries, partner nations, and international aid organizations for such interventions is crucial to improve outcomes in this vulnerable population.

Additionally, prostate cancer awareness among this population is another important target for improvement. Prior studies have demonstrated that cancer awareness among refugee populations is low, and this low awareness translates into reduced use of screening and more advanced cancer stage at diagnosis (15, 26–28). Practical methods for increasing prostate cancer awareness in refugee populations have been previously studied, and include native language patient education materials with good readability, increased availability of community-based (rather than clinic-based) practitioners, and using trained community individuals (rather than medical professionals) to help disseminate health- and cancer-related education (29, 30).

An additional consideration when delivering medical treatment to low-resource populations such as refugee populations is the informed consent process. Factors common in refugee populations, such as poverty, lack of formal education, and reduced literacy all contribute to challenges in obtaining a truly informed consent when presenting a treatment option to a patient (31). For example, a study of low-income, low-literacy patients with advanced cancer in Mexico revealed that half of patients found consent forms for cancer treatment difficult or impossible to understand (32).

## Limitations

The present study has several limitations. Information regarding the method of initial diagnosis (i.e., asymptomatic screening versus symptom-driven presentation) were not available. This information would be valuable to aid understanding of the use of prostate cancer screening in refugee populations. Only limited clinical and oncologic data were available regarding patients’ diagnosis and treatment. Reasons and rationale for physician decision-making with regard to dose-fractionation schedules were not available. It is likely that a proportion of patients treated with palliative intent presented with spinal cord compression or other complex metastases for which a protracted palliative course may have been favored; however, it is reasonable to assume that many patients treated with palliative intent had simple bone metastases. Similarly, physicians’ rationale for the omission of ADT was also not available. The present study focused on patients in settings with a higher concentration of refugee patients; the identified trends may not be generalizable to patterns of care in settings with fewer refugee patients.

## Conclusion

In the first study to date of prostate cancer diagnosis and utilization of radiation therapy in refugee patients, prostate cancer was overwhelmingly diagnosed at an advanced stage. Most patients were not treated with internationally-accepted standards of care regarding androgen deprivation therapy. Furthermore, hypofractionated radiotherapy was not utilized in definitive treatment courses. The treatment compliance rate was low, likely reflecting significant barriers to care faced by this population, magnified by protracted on-treatment time. Interventions to improve screening in this patient population and to increase the use of standard-of-care treatment paradigms, including hypofractionated RT, in physicians treating this population are critically needed.

## Data availability statement

The original contributions presented in the study are included in the article/supplementary material, further inquiries can be directed to the corresponding author.

## Ethics statement

The studies involving human participants were reviewed and approved by Institutional Review Boards of Marmara University Pendik Research and Education Hospital (IRB# 09.2019.615). Written informed consent for participation was not required for this study in accordance with the national legislation and the institutional requirements.

## Author contributions

All authors listed have made a substantial, direct, and intellectual contribution to the work and approved it for publication.

## Conflict of interest

The authors declare that the research was conducted in the absence of any commercial or financial relationships that could be construed as a potential conflict of interest.

## Publisher’s note

All claims expressed in this article are solely those of the authors and do not necessarily represent those of their affiliated organizations, or those of the publisher, the editors and the reviewers. Any product that may be evaluated in this article, or claim that may be made by its manufacturer, is not guaranteed or endorsed by the publisher.
